# A parametric study on the decolorization and mineralization of C.I. Reactive Red 141 in water by heterogeneous Fenton-like oxidation over FeZSM-5 zeolite

**DOI:** 10.1186/s40201-015-0162-6

**Published:** 2015-01-31

**Authors:** Ceyda Yaman, Gönül Gündüz

**Affiliations:** Chemical Engineering Department, Ege University, 35100 Bornova, İzmir, Turkey

**Keywords:** Reactive red 141, Heterogeneous Fenton-like oxidation, Fe-ZSM-5 zeolite catalyst

## Abstract

In this study, the heterogeneous Fenton-like degradation of Reactive Red 141 (RR141) in water was investigated over iron containing ZSM-5 zeolite (Si/Al = 42) prepared by ion-exchange (FeZSM-5 (42)). The catalyst was characterized by XRD, SEM, FTIR, TPR, and ICP-AES measurements. The effects of the initial concentrations of the dye and H_2_O_2_, the initial pH of the solution, catalyst loading, and the reaction temperature were investigated on heterogeneous Fenton-like degradation of RR141. The reduction of the solution initial pH from ca. 7.0 to ca. 3.5 had a positive effect on color removal. A complete color removal was achieved with all the H_2_O_2_ concentrations in the range of 0.007 - 0.067 M over the FeZSM-5 (42) catalyst after 2 h of reaction. The COD reduction increased from 52% to 81% with an increase in the amount of the catalyst. The COD reduction was affected positively with the increase in temperature from 298 K through 313 K to 323 K and then to 333 K. The initial decolorization rate was described by the equation: −r_A0_ = 9.4*10^4^ e^-38.8/RT^ C_RR141,0_ C_H2O2,0_^0.184^ with an H_2_O_2_ concentration range of 0.007 M – 0.033 M (where R is in kJ/mol).

## Background

Sulfonated and unsulfonated azo dyes have a negative aesthetic effect on the wastewater that is highly colored even at a low concentration of dye. On the other hand these dyes are toxic, carcinogenic and mutagenic. It is difficult to remove azo dyes in wastewater produced by textile industries using biological, physical and chemical treatment methods because of the chemical stability of these dyes, the high cost of the processes, and also disposal problems. Therefore, it is necessary to find an effective method for the wastewater treatment of reactive azo dyes. In the last decade, attention has focused on the Advanced Oxidation Processes (AOP) that is based on the generation of highly reactive hydroxyl radicals.

Among them, heterogeneous Fenton’s reaction is very promising because it is a cost-effective method with a high reaction yield and regeneration of the catalyst is possible. The removal of the catalyst from the purified water is easy and a narrow range of pH values at which the reaction occurs is not required. On the other hand, leaching of iron ions from the solid catalyst is limited [1-16]. Heterogeneous Fenton-type catalysts have been developed by incorporating transitional metal cations such as iron ions or iron oxides into porous supports such as zeolites and pillared clays [17-21]. In the presence of H_2_O_2_, hydroxyl radicals (OH^.^) are produced by interaction of H_2_O_2_ with the iron (II) species present on the porous support. The main reaction mechanism of heterogeneous Fenton process is described as follows [22-26]:

1 $$ \mathrm{X}\hbox{-} {\mathrm{Fe}}^{2+} + {\mathrm{H}}_2{\mathrm{O}}_2\to \mathrm{X}\hbox{-} {\mathrm{Fe}}^{3+}+{\mathrm{O}\mathrm{H}}^{\hbox{-} } + \mathrm{H}\mathrm{O} $$

where X presents the surface of the catalyst. Iron (III) can then react through Equations 2–4 with hydrogen peroxide in order to regenerate iron (II) supporting the Fenton process:

2 $$ \mathrm{X}\hbox{-} {\mathrm{Fe}}^{+3} + {\mathrm{H}}_2{\mathrm{O}}_2\rightleftarrows \mathrm{X}\hbox{-} {\mathrm{Fe}\mathrm{OOH}}^{2+}+{\mathrm{H}}^{+} $$

3 $$ \mathrm{X}\hbox{-} {\mathrm{Fe}\mathrm{OOH}}^{2+}\to {{\mathrm{HO}}_2}^{.} + \mathrm{X}\hbox{-}\ {\mathrm{Fe}}^{2+} $$

4 $$ \mathrm{X}\hbox{-} {\mathrm{Fe}}^{3+} + {\mathrm{H}\mathrm{O}}_2\to \mathrm{X}\hbox{-} {\mathrm{Fe}}^{2+} + {\mathrm{O}}_2 + {\mathrm{H}}^{+} $$

The hydroperoxyl radicals (HO_2_^.^) generated in Eq.3 are less reactive than the OH^.^ species. In the application of heterogeneous Fenton-like oxidation to dye degradation, the following reactions take place: Firstly, the formed oxidative radicals (OH^.^ and HO_2_^.^) react with the activated dye molecules to produce intermediate products and then mineralization products (H_2_O + CO_2_) through Eqs. 5–6:

5 $$ \mathrm{X}\hbox{-} {\mathrm{Fe}}^{3+} + \mathrm{dye}\to \mathrm{X}\hbox{-} {\mathrm{Fe}}^{2+}{+}^{.}{\mathrm{dye}}^{+} $$

6 $$ \mathrm{Oxidative}\ \mathrm{radicals}\ {+}^{.}{\mathrm{dye}}^{+}\to \mathrm{Intermediate}\ \mathrm{products}\to {\mathrm{CO}}_2 + {\mathrm{H}}_2\mathrm{O} $$

Decolorization of the dye is a result of N = N bond destruction with the addition of OH^.^. The mineralization of dye is the total oxidation of the intermediate products to CO_2_ and H_2_O and it is slower than color removal because of the priority of the OH^.^ attack on the N = N bonds [1].

**Figure 1:**
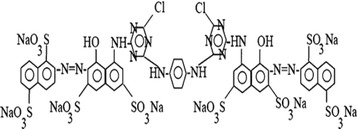
**Chemical structure of reactive red 141.**

C.I. Reactive Red 141 (RR141) is a sulfonated, bright red color diazo reactive textile dye and is widely used for the dyeing processes in the textile industry. Procion Red H-E7B and C.I. Reactive Red 141 are the commercial names of the dye. Its empirical formula is C_52_H_22_Na_8_O_26_S_8_C_l2_N_14_ and is composed of two monochlorotriazine reactive groups. It has a molecular weight of 1774 g/mol and its chemical structure is given in Figure 1. The adsorption spectra of RR141 is characterized by two main bands, one in the visible region (λmax = 543 nm) and the other in the UV region (λmax = 288 nm). In the heterogeneous Fenton-like oxidation of RR141, hydroxyl radical attack on the N = N bonds occurs at 543 nm and this band is responsible for the chromophoric components in the dye.

The degradation of RR141 has been investigated by biodegradation [27-29], by adsorption [22,30,31], by homogeneous Fenton’s reaction, by non-catalytic or catalytic photooxidation [32-41], by electrochemical oxidation with conductive-diamond anode [42], and by ultrasound [1]. Few studies have been reported on the heterogeneous Fenton-like degradation of C.I. Reactive Red 141 to date. In one study, iron oxide particles recovered from acid mine drainage were used as an adsorbent or as a catalyst in the oxidation of RR141 through a Fenton-like mechanism to remove the dye from the aqueous solution [22]. However, the main aim of that study was to investigate the dye-adsorption capacity of iron oxide rather than its catalytic effect in the heterogeneous Fenton-like oxidation of dye. Only one experiment has been carried out for dye degradation by heterogeneous Fenton’s reaction and a TOC removal of around 3% could only be achieved.

Conventional processes such as adsorption, coagulation/flocculation (CF) etc., may be costly, ineffective and produce a high amount of secondary wastes. CF is still not good enough to be used in industry even when it is combined with microfiltration or ultrafiltration. However, catalytic methods such as heterogeneous Fenton-like oxidation result in permanent chemical degradation of dyes. This process is still simple, non-expensive, a promising, and attractive treatment method for the effective decolorization and degradation of dyes when compared with the conventional processes [25-43].

Many studies are found in literature on the removal of dyes using Fe-ZSM-5 [3,23,24,44], Fe-Y [2,25,45-48], and Fe-clay [26,49-52] catalysts by several advanced oxidation techniques. The catalytic activity and stability of these catalysts depend on the synthesis method in addition to their composition and framework structure. The leakage of iron ions from the support is the other important factor to be considered in dye degradation over an iron containing support: because iron leaching from the catalyst causes a new pollution of the treated water. The studies showed that iron containing ZSM-5 zeolite exhibited better catalytic activity and stability than iron-containing Y zeolites. On the other hand, the activity of iron-containing clay catalysts in the degradation of dyes depends on the careful selection of the preparation method and calcination conditions. However, till now no a detailed parametric study including decolorization kinetics has not been reported on the heterogeneous Fenton-like degradation of dyes on iron containing ZSM-5 zeolites. The objectives of this study are to: (1) investigate the influence of the reaction conditions of the heterogeneous Fenton-like oxidation and optimize them, (2) investigate the decolorization kinetics, and (3) investigate the leakage of iron ions in the reaction for the stability of the catalyst.

Our group previously published a study [53] on the degradation of RR141 using the heterogeneous Fenton-like process over iron containing ZSM-5 zeolites. That work included the preparation of the catalysts by ion-exchange or hydrothermal synthesis and their characterization studies by XRD, SEM, FTIR, ICP-AES, TPR, and nitrogen adsorption. The catalytic activity screening tests of the prepared catalysts were accomplished to determine the catalyst with the highest activity in the degradation of RR141 by heterogeneous Fenton-like oxidation reaction. The highest color removal (97%) was achieved by the catalyst prepared by ion exchange with a silicon/aluminum ratio of 42 zeolite (FeZSM-5(42)). The above mentioned objectives of the presented study was investigated over that iron-containing ZSM-5 zeolite catalyst. The presented study is novel and unique from these points of view.

## Methods

### Preparation of the catalysts

Ion exchange was used for the preparation of the iron containing the ZSM-5 zeolite catalyst. The method of Schwidder et al. [54] was applied with minor modifications [21] for the ion-exchange. The ZSM-5 zeolite with silicon/alumina = 42 was obtained from Süd-Chemie AG (Germany). An iron exchange of 98.9% could be achieved. The catalyst was coded as FeZSM-5 (42).

### Catalyst characterization

The prepared catalyst was characterized by nitrogen adsorption, X-ray diffraction patterns (XRD), scanning electron microscope (SEM), Fourier transform-infrared (FTIR) spectrometer, inductively coupled plasma atomic emission spectrometer (ICP-AES), and by temperature programmed reduction (TPR) measurements. The precise procedures for the characterization of the catalyst are described in Ref [53].

### Heterogeneous Fenton-like oxidation of RR141

**Figure 2:**
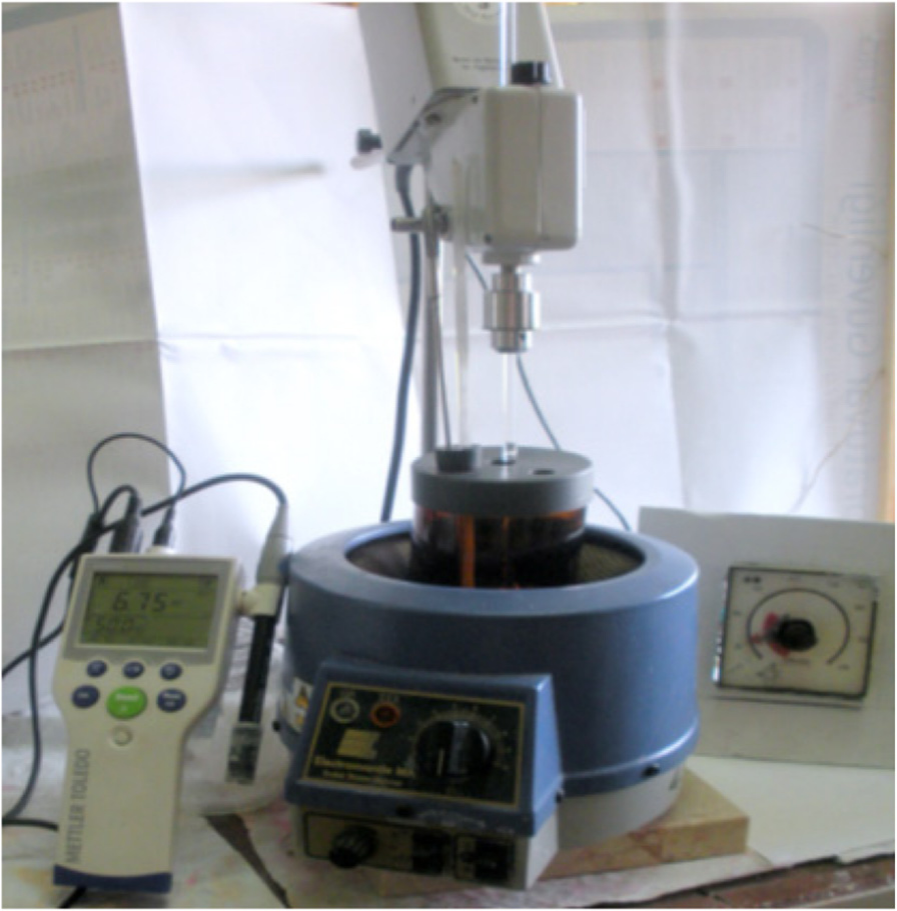
**Experimental set-up.**

C.I. Reactive Red 141 (Procion Red H-E7B) was purchased from Dystar (Germany) with a dye content of >85% and was used as received without further purification. The heterogeneous Fenton-like degradation of RR141 was carried out under isothermal conditions in a temperature controlled shaded glass batch reactor with a volume of 470 mL equipped with a mechanical stirrer at ca. 280 rpm (Heidolph, Germany) and a pH electrode (Mettler Toledo). Figure 2 presents the picture of the experimental set-up. In a typical run, 0.15 dm^3^ of aqueous dye solution (0.1 g-dye/dm^3^-soln = 5.64×10^−5^ M) was placed into the reactor and the temperature was adjusted to 333 K. When the temperature reached to 333 K, the pH of the dye solution was measured and 0.15 g of catalyst (1 g-cat/dm^3^ –soln.) was introduced into the solution under continuous stirring. After the adsorption equilibrium was established (roughly in 10–15 min) the solution was analyzed to determine the dye removal by adsorption. Then a solution of 35% H_2_O_2_ (Merck, Germany) (40 mmol/0.15 dm^3^ solution, namely 0.267 M) was added to the dye solution. After the addition of H_2_O_2_, the pH of the solution was again measured. This was recorded as the starting time of the reaction. The samples taken periodically were centrifuged for 0.5 h to remove the catalyst and kept at 273 K to stop the reaction before the analysis with the UV spectrophotometer (Jasco 7800 UV/vis).

The decrease of the intensity of the band at 543 nm was used as a measure of decolorization degree. The reduction in the chemical oxygen demand (COD) of the dye solution was determined using a Lovibond (Germany) Checkit Direct COD Vario device for each run after a reaction time of 2 h.

The blank run with the above conditions, but without H_2_O_2_ showed that dye removal by adsorption was not greater than 3% and 21% in 15 min and 120 min of reaction, respectively. A color removal of 11% could be achieved in the presence of H_2_O_2_ only in 2 hour of reaction.

Each experiment was conducted in duplicate and the standard deviation of the average of the independent runs for color removal changed in the range of ±0.51 to ±1.29.

## Results and discussion

### Catalyst characterization studies

The X-ray diffraction patterns of the FeZSM-5 catalyst sample showed the typical diffractrograms of the MFI structure (2θ = 7-9^0^ and 23-25^0^). The incorporation of iron in the MFI lattice did not damage the zeolite structure [53].

**Figure 3:**
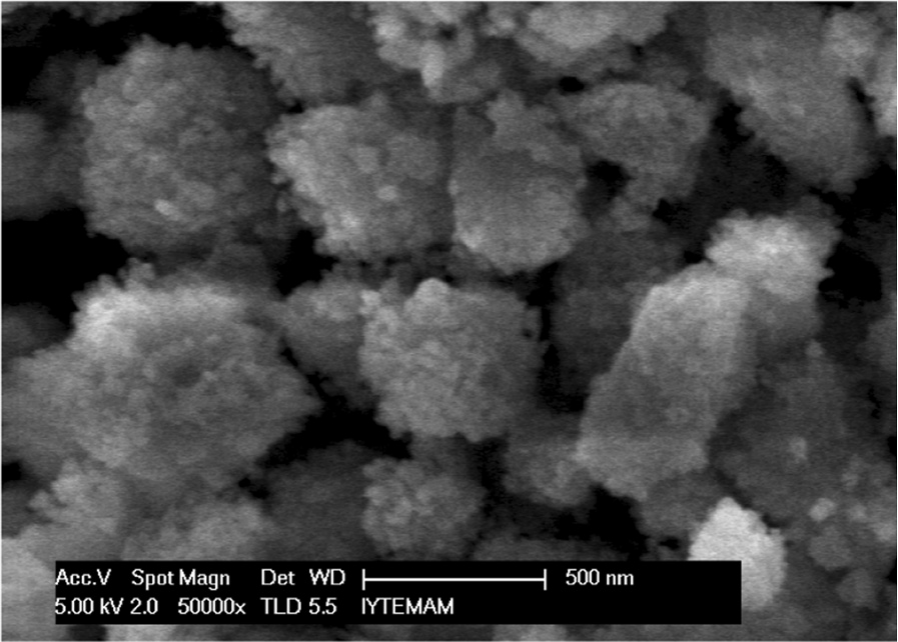
**SEM image of the catalyst.**

The SEM images of the catalyst sample depicted that the crystallites in the FeZSM-5 (42) sample were a spherical shape (Figure 3).

Iron content and Si/Al ratio of the catalyst were determined with the ICP-AES method and found to be 4.98 wt% and 42.8, respectively. The BET surface area, total pore volume, average pore diameter and micropore area of the catalyst were found to be 469 m^2^/g, 0.321 cm^3^/g, 0.73 nm (using the Horvath-Kawazoe method), and 236 m^2^/g, respectively.

The FTIR spectra of the catalyst showed bands at 450, 550, 800, 1100, 1225, and 1650 cm^−1^ which were assigned to different vibrations of the tetrahedral and framework structure of the ZSM-5 zeolite [53].

The temperature-programmed reduction with hydrogen (H_2_-TPR) of the catalyst showed that a major hydrogen consumption peak was obtained at 397 K which reflected the reduction of out of framework iron oxides [53].

### Influence of the initial pH of solution on the degradation of dye

The effect of solution pH on the degradation of dye was investigated at an initial dye pH of ca. 7 and at a pH of 3.5 which was regulated by the addition of 0.1 N sulphuric acid to the dye solution.

**Figure 4:**
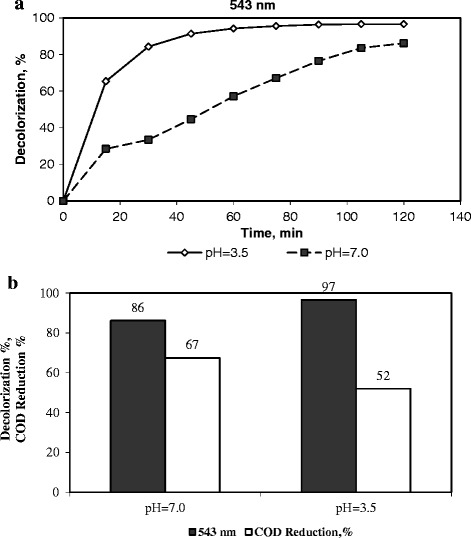
**The influence of the initial pH of the solution on the degradation of RR141.** (Initial dye concentration = 0.1 g/dm^3^, catalyst loading 1 g-cat/dm^3^-soln, H_2_O_2_ concentration = 0.267 M, and temperature = 333 K). **a)** Decolorization as a function of time, **b)** Decolorization, %, and COD removal, %, after a reaction duration of 2 h.

Figure 4 shows the effect of the solution pH on the color and COD removals for RR141 degradation with the catalyst. The reduction of pH to around 3.5 positively affected the color removal. The acidic environment increased the decolorization degree of the dye from 86% to 97% after a reaction duration of 2 h. The pKa of the sodium sulfonate groups of the dyes is very low (<1), therefore, the RR141 is expected to exist as anionic groups (−SO_3_^−^) at a pH > 1.0. On the other hand, the interaction between the anionic groups of dye and surface acid sites of the Fe-ZSM-5 zeolite catalysts becomes stronger at a low pH. The observed increase in decolorization at a low pH can be attributed to a greater adsorption of dye on the catalyst surface and consequently it results in a higher decolorization of the dye [40]. However, a decrease in COD removal from 67% to 52% was observed with the regulation of the solution pH to a ca. 3.5. This may be due to the formation of intermediates such as monoazo, naphthalene, and naphthol disulfonic acid intermediates whose degradation is hindered at acidic pH [20,29].

The amount of iron loss from the catalyst into the solution was determined by measuring the iron concentration in the solution after a reaction duration of 2 h, with an atomic absorption spectrophotometer (Varian 10 plus). The leaching of iron cations from the zeolite into the solution depended strongly on the pH [4]. As expected, with the regulation of pH from 7 to about 3.5 the iron leaching increased from 0.3×10^−3^ g/dm^3^ to 0.9×10^−3^ g/dm^3^. It means that iron loss changed from 0.59% to 1.77%. As seen, the iron leaching was considerably low (being below the EU directives of < 2×10^−3^ g/dm^3^).

### Influence of the hydrogen peroxide concentration on the degradation of dye

**Figure 5:**
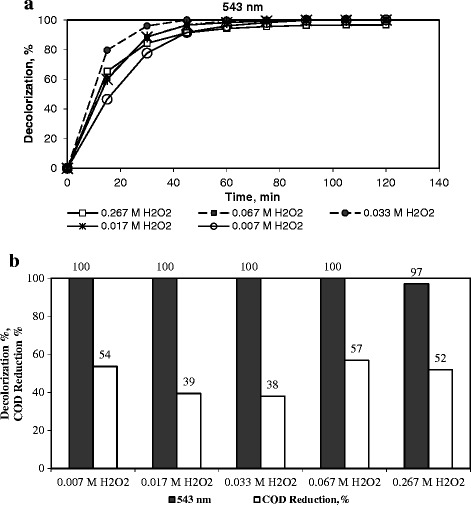
**The influence of the initial H**_**2**_ **O**_**2**_ **amount on the degradation of RR141.** (Initial dye concentration = 0.1 g/dm^3^, catalyst loading 1 g-cat/dm^3^-soln, initial pH ~ 3.5, and temperature = 333 K). **a)** Decolorization as a function of time, **b)** Decolorization, %, and COD removal, %, after a reaction duration of 2 h.

The effect of the H_2_O_2_ concentration on the degradation of RR141, while maintaining constant all the other operating parameters (catalyst loading: 1 g-cat/dm^3^–soln., initial pH ~ 3.5, initial concentration of dye = 0.1 g-dye/dm^3^-soln., temperature = 333 K) was investigated and is given in Figure 5. The increase in H_2_O_2_ concentration from 0.007 M to 0.017 M and to 0.033 M positively affected the initial rate of color removal (Figure 5a.). This result is expected because of the increase of the OH^.^ radicals produced with the increasing amount of H_2_O_2_, Eq.1. Nevertheless the increase in H_2_O_2_ concentration from 0.033 M to 0.067 M and to 0.267 M slowed the initial decolorization of RR141. A complete color removal was achieved with all the initial concentrations of H_2_O_2_ studied, except with 0.267 M (97%) after 2 h of oxidation. The slight decrease in the initial decolorization of RR141 with an increasing H_2_O_2_ can be attributed to the reaction between the H_2_O_2_ and generated hydroxyl radicals to produce less reactive hydroperoxyl radicals (HO_2_^.^):

7 $$ {\mathrm{H}}_2{\mathrm{O}}_2+\mathrm{O}\mathrm{H}\to {{\mathrm{H}\mathrm{O}}_2}^{.} + {\mathrm{H}}_2\mathrm{O} $$

The dominant response from the two opposite effects given in Equations 1 and 7 directs the reaction. Similar results have been reported by Neamtu et al. [2] and Ramirez et al. [4], in the Fenton-like oxidation of Procion Marine H-EXL and Orange II, respectively.

Figure 5b presents the percentages of decolorization and COD removal in the degradation of RR141 after a reaction time of 2 h. COD reduction reached 57% in the presence of 0.067 M of H_2_O_2_, while it was 54% at a dose of 0.007 M H_2_O_2_ and decreased to 38% with the increase in H_2_O_2_ concentration from 0.007 M to 0.033 M. A COD removal of 52% was obtained at a dose of 0.267 M H_2_O_2_. The COD removal is significantly lower than the color removal at all doses of H_2_O_2_. This result signifies the priority of the OH^.^ attack on the N = N bonds and the slower destruction of the aromatic/olefinic carbons in the dye [1]. For cost considerations, the optimum H_2_O_2_ concentration can be selected as 0.007 M.

In literature, the COD (chemical oxygen demand), TOC (Total Organic Carbon), and DOC (dissolved organic carbon) removals were measured in the degradation of RR141 by several advanced oxidation techniques. For instance a TOC removal of about 3% was achieved in the heterogeneous Fenton-like oxidation of RR141 over iron oxides particles recovered from acid mine drainage [22].

A 17%-23% TOC removal was achieved in the degradation of RR141 by the ferrioxalate-Fenton/UV-A and TiO_2_/UV-A processes [32].

A 100% DOC removal was obtained for RR141 after a 90 min treatment under homogeneous Fenton assisted solar light [34].

A decrease of 64% in COD was reached in the degradation of RR141 within 5 hours using the UV/H_2_O_2_ advanced oxidation process [37].

COD was reduced from 120 mg/L to 23 mg/L (81%) in the solar nano-photocatalytic degradation of RR141 using TiO_2_ [38].

A 37% removal of TOC was achieved in the photocatalytic degradation of RR141 for the Zn_2_SnO_4_ photocatalyst after 270 min of sunlight irradiation [39].

A TOC reduction of 29% was achieved in the degradation of RR141 with a TiO_2_ coated pebble bed photocatalytic reactor having a solar collector [40].

A COD removal of 100% was obtained in the electrolyse of an aqueous solution of RR141 with a conductive-diamond anode [42].

### The influence of the catalyst amount on the degradation of dye

**Figure 6:**
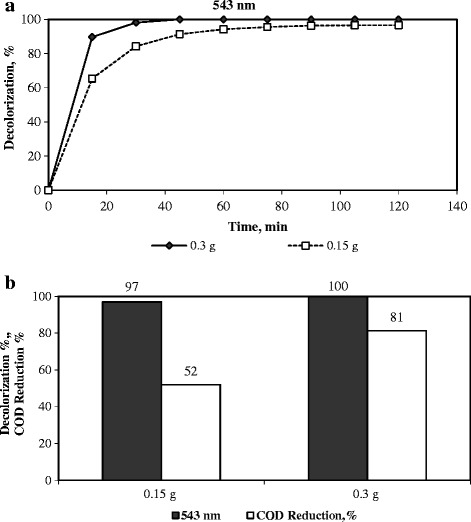
**The influence of the catalyst amount on the degradation RR141.** (Initial dye concentration = 0.1 g/dm^3^, initial pH = 3.5, H_2_O_2_ concentration = 0.267 M, and temperature = 333 K). **a)** Decolorization as a function of time, **b)** Decolorization, %, and COD removal, %, after a reaction duration of 2 h.

The effect of the catalyst amount was investigated on the Fenton-like degradation of RR141 using catalyst amounts of 0.15 g and 0.3 g for 0.150 dm^3^ dye solution under the following reaction conditions: an initial RR141 concentration of 0.1 g/dm^3^, a temperature of 333 K, an initial pH = 3.5, an initial H_2_O_2_ concentration = 0.267 M. The results are shown in Figure 6. Doubling the amount of catalyst slightly increased the color removal from 97% to 100% after 2 hours of reaction. The COD reduction increased from 52% to 81% with an increase in the amount of the catalyst. This remarkable increase in COD removal may be attributed to the production of more hydroxyl radicals in the degradation reaction of RR141. Because increasing the catalyst amount provides more active catalytic sites of Fe^2+^/Fe^3+^ species to enhance degradation. A similar trend where the catalyst amount influences the degradation of dye was reported in literature, as well [2,4,20].

### Influence of the temperature on the degradation of dye

**Figure 7:**
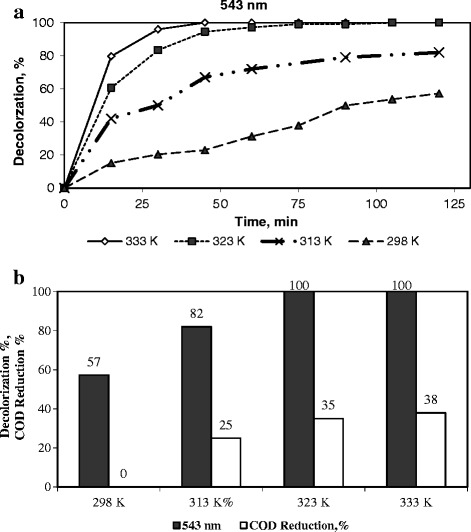
**The influence of temperature on the degradation RR141.** (Initial dye concentration = 0.1 g/dm^3^, initial pH = 3.5, H_2_O_2_ concentration = 0.033 M, and catalyst loading 1 g-cat/dm^3^-soln). **a)** Decolorization as a function of time, **b)** Decolorization, %, and COD removal, %, after a reaction duration of 2 h.

Experiments were conducted to investigate the effect of temperature on the degradation of an aqueous dye solution at four different temperatures, 298, 313, 323, and 333 K under the following conditions: an RR141 initial concentration of 0.1 g/dm^3^, a catalyst amount of 0.15 g/0.150 dm^3^ dye solution, an initial pH of 3.5, and an H_2_O_2_ amount of 0.033 M (at which the initial decolorization rate was the highest). The results are presented in Figure 7. It was clearly seen that the color removal rate increased with the increase in temperature from 298 K through 313 K to 323 K and then to 333 K. These results agree with the fact that the Fenton reaction was accelerated with the increasing temperature, describing an Arrhenius behavior [33]. Because both the collision frequency of the molecules at the surface of the catalyst and the fraction of molecules that possess energy in excess of the activation were increased. Temperature has a significant role at enhancing the extent of the decolorization of dye with the generated OH^.^, since it is a thermodynamic state function that enhances the feasibility of a chemical process [26]. The lowest degradation degree was measured at a temperature of 298 K. Furthermore, while the color removal increased with the increase in temperature no COD reduction was observed at 298 K, however the COD reduction was enhanced with the temperature being 25%, 35%, and 38% at temperatures higher than 298 K, respectively. Regarding the time required to achieve, for instance 40% of RR141 degradation, it was found that this time significantly decreased when the temperature was increased, with t = 78.0, 14.5, 10.0, and 7.5 min at 298, 313, 323, and 333 K, respectively.

In literature, similar results have been reported in the catalytic wet peroxide oxidation (CWPO) of Orange II over an Fe-saponite catalysts [4], in the CWPO of phenol over pillared clays containing iron [55] and in the wet oxidation of 2.4.6-trichlorophenol in water using an Fe^3+^, Co^2+^, Ni^2+^ supported MCM-41 catalysts [18].

### Stability of the catalyst

**Figure 8:**
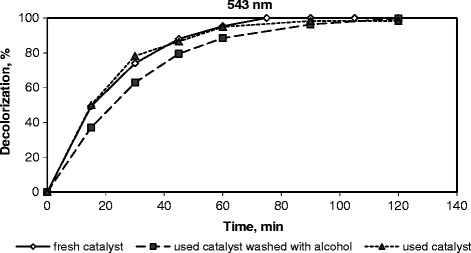
**Stability of the catalyst in the oxidation of RR141.** (Initial dye concentration = 0.1 g/dm^3^, initial pH = 3.5, H_2_O_2_ concentration = 0.033 M, catalyst loading 1 g-cat/dm^3^-soln, and temperature = 333 K).

For the stability studies, the runs were carried out using an 0.15 dm^3^ of 0.1 g/dm^3^ RR141 aqueous solution with an addition of 0.033 M H_2_O_2_ at 333 K, and at a pH of 3.5. To recover the catalyst, after 2 h of oxidation the final effluent was filtrated and dried or washed with ethanol and then water. These catalysts were labeled as used catalyst and used catalyst washed with alcohol, respectively. The latter one was calcined by heating to 423 K and holding there for 15 min then heating to 873 K and keeping at this temperature for 2 h to remove the adsorbed organic species from the active sites [4,56] and their performances reached in terms of RR141 decolorization are presented in Figure 8. The same color removal results were obtained after 2 h oxidation of RR141 over fresh, used and used catalyst washed with alcohol. A slight decay in the initial decolorization rate was obtained only on the used catalyst washed with ethanol. The loss of activity can be attributed to the poisoning of the active catalytic sites due to the adsorbed organic species or oxidation of Fe^2+^ to Fe^3+^.

In literature, similar results were reported in the studies on the CWPO of Orange II over Fe-saponite catalysts [4] and on CWPO of propionic acid over a FeZSM-5 catalyst [57].

### Decolorization kinetics of the Fenton-like oxidation of RR141

In the Fenton-like oxidation of RR141, the reaction mixture was stirred vigorously at around 280 rpm. The external diffusion effects were calculated using Hougen’s criterion and it was found that (C_b_-C_s_)/C_b_ ≈ 0.04, so it was assumed that C_b_ ≈ C_s._ The internal diffusion resistance was negligible due to the small size of the catalyst particles (500 nm). To calculate the internal diffusion effects, the generalized Thiele modulus based on the reaction rate was determined and found to be 0.339×10^−6^ and hence the effectiveness factor was assumed to be unity [58].

The decolorization kinetics was determined at an initial pH of 3.5 using the initial decolorization rates and a first order dependency was obtained on the dye concentration with R^2^ = 1. This result was in good agreement with those reported in literature for decolorization kinetics of RR141 degraded by several advanced oxidation techniques [1,32,34,37,38,40,59]. However, the photo-catalytic degradation of RR141 on Zn_2_SnO_4_ followed the zero order kinetics under sunlight [41].

**Figure 9:**
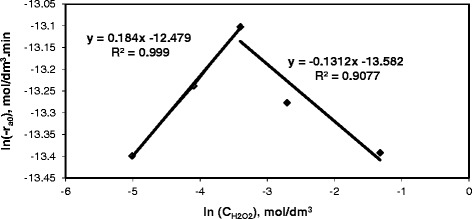
**The variation of the initial rate with respect to the initial concentration of H**_**2**_ **O**_**2.**_

The order with respect to H_2_O_2_ concentration was determined by plotting the initial rate, −ln(−r_A0_), against the initial H_2_O_2_ concentration, ln(C_H2O2,0_). Figure 9, presents the variation of the initial rate with respect to the initial concentration of H_2_O_2_ for 0.1 g/dm^3^ dye concentration at 333 K.

As it is seen in Figure 9, there is a break at the H_2_O_2_ concentration of 0.033 M. Namely, order of reaction depends on the amount of H_2_O_2_ (shifting-order reaction). The order of reaction is 0.184 with respect to H_2_O_2_ in the range of 0.007 M-0.033 M H_2_O_2_ and −0.131 with respect to H_2_O_2_ greater than 0.033 M H_2_O_2_. The initial rate is expressed by equations (8) and (9) based on the amount of H_2_O_2_.

8 $$ \hbox{-} {\mathrm{r}}_{\mathrm{A}0} = \mathrm{k}\ {\mathrm{C}}_{\mathrm{RR}141,0}{{\mathrm{C}}_{\mathrm{H}2\mathrm{O}2,0}}^{0.184}\kern1.4em \left(0.007\hbox{-} 0.033\ \mathrm{M}\right) $$

9 $$ \hbox{-} {\mathrm{r}}_{\mathrm{A}0} = \mathrm{k}\ {\mathrm{C}}_{\mathrm{RR}141,0}{{\mathrm{C}}_{\mathrm{H}2\mathrm{O}2,0}}^{\hbox{-} 0.131\kern5em }\kern-2em \left(>0.033\right) $$

In these equations, −r_A0_ has the units of mol/dm^3^ min, and C_RR141,0_ and C_H2O2,0_ are in units of mol/dm^3^, and k is the reaction rate constant. As seen from Equation 7, at high H_2_O_2_ concentrations it reacts with OH^.^ radicals to generate less reactive perhydroxyl radicals, HO_2_^.^, causing a decrease in the color removal rate. Table 1 shows the reaction rate constant, k, calculated at different temperatures (298, 313, 323, and 333 K) by using the initial rate equation (8).

**Table 1:** **Reaction rate constants at different temperatures**

**Temperature (T), K**	**-r**_**A0**_ **(mol/dm** ^**3**^ **.min)**	**k (dm** ^**3**^ **/mol)** ^**0,184**^ **min** ^**−1**^	**C**_**RR141**_ **(mol/dm** ^**3**^ **)**	**C**_**H2O2**_^**0,184**^ **(mol/dm** ^**3**^ **)** ^**0.184**^
333	2.041*10^−6^	0.0677	5.64*10^−5^	0.535
323	1.708*10^−6^	0.0567	5.64*10^−5^	0.535
313	1.122*10^−6^	0.0328	5.64*10^−5^	0.535
298	4.171*10^−6^	0.0138	5.64*10^−5^	0.535

**Figure 10:**
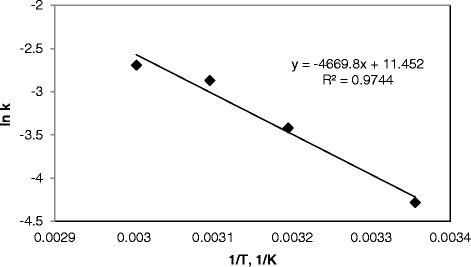
**lnk versus 1/T dependency for the decolorization of RR141.**

Figure 10 presents the Arrhenius plot of lnk vs. 1/T obtained by using the data in Table 1. From the slope of the Arrhenius plot (R^2^ = 0.97) in Figure 10, −E/R, where R is the universal gas constant (8.314 J/mol K), and the activation energy, E, was calculated to be 38.8 kJ/mol. Finally, the initial decolorization rate of RR141 can be expressed by the following equation for the range of 0.007 M - 0.033 M H_2_O_2_;

10 $$ \hbox{-} {\mathrm{r}}_{\mathrm{A}0}=9.4*{10}^4{\mathrm{e}}^{\hbox{-} 38.8/\mathrm{R}\mathrm{T}}{\mathrm{C}}_{\mathrm{RR}141,0}{{\mathrm{C}}_{\mathrm{H}2\mathrm{O}2,0}}^{0.184} $$

In literature, the activation energy for the heterogeneous Fenton-like reaction of RR141 has not been declared. This study is a useful reference in the decolorization kinetics of RR141 for the design of RR141 removal reactors. In the homogeneous photo-Fenton reaction of RR141, activation energy was estimated to be 1.26 kJ/mol [34]. The lack of mass transfer resistances facilitates the homogeneous catalytic reactions with low activation energies. Decomposition of RR141 with the ferrioxalate-Fenton/UV-A process was fitted to the empirical Langmuir-Hinshelwood kinetic model [32]. An activation energy of 35.9 kJ/mol was reported for the rapid decolorization of azo dye methyl orange in an aqueous solution by nanoscale zerovalent iron particles [60]. This value is very close to the activation energy (38.8 kJ/mol) obtained in this study for the heterogeneous Fenton-like oxidation of Reactive Red 141. Moreover, in literature, the activation energy of the homogeneous catalytic Fenton oxidation of Reactive Brillant Blue X-BR azo dye was given to be 25.21 kJ/mol [61].

## Conclusions

The heterogeneous Fenton-like degradation of Reactive Red 141 (RR141) in water was investigated over iron containing ZSM-5 zeolite (Si/Al = 42) prepared by ion-exchange. The catalyst seems to have a promising efficiency in RR141 degradation. A complete color removal could be achieved with the catalyst at a pH of 3.5 in an H_2_O_2_ concentration range of 0.007 M-0.067 M after two hours of reaction. The increase in H_2_O_2_ concentration up to 0.033 M increased the initial decolorization due to the increase of the OH^.^ radicals formed. However, at H_2_O_2_ concentrations higher than 0.033 M the initial decolorization rate of RR141 was decelerated. The highest COD removal was obtained to be 81% with a complete color removal at an H_2_O_2_ concentration of 0.267 M and at 333 K with a catalyst amount of 0.3 g for 0.15 dm^3^ a dye solution of 0.1 g/dm^3^. An increase in temperature positively affected the decolorization of dye as well as the COD removal. The initial decolorization rate was described as -r_A0_ = 9.4*10^4^ e^-38.8/RT^ C_RR141,0_ C_H2O2,0_^0.184^ in the H_2_O_2_ concentration range of 0.007 M – 0.033 M. Iron leaching remained below the EU directives. The small iron leaching makes it possible for the catalyst to have long term stability without generating iron hydroxide sludge.

It can be concluded that this study offers a significant potential for the application of heterogeneous Fenton-like oxidation over iron containing a ZSM-5 zeolite for the degradation of RR141 aqueous solutions. It will be a good alternative to the oxidation methods currently used in the degradation of RR141.
